# The Assessment of the Reliability and Validity of the Polish Version of the Adult Vaccine Hesitancy Scale (PL-aVHS) in the Context of Attitudes toward COVID-19 Vaccination

**DOI:** 10.3390/vaccines10101666

**Published:** 2022-10-06

**Authors:** Mariusz Duplaga, Urszula Zwierczyk, Kinga Kowalska-Duplaga

**Affiliations:** 1 Department of Health Promotion and e-Health, Faculty of Health Sciences, Institute of Public Health, University Medical College, 30-611 Krakow, Poland; 2 Department of Pediatrics, Gastroenterology and Nutrition, Faculty of Medicine, Jagiellonian University Medical College, 30-663 Krakow, Poland

**Keywords:** vaccination, vaccine hesitancy, adult vaccine hesitancy scale, health literacy, COVID-19, confirmatory factor analysis, exploratory factor analysis, reliability, validity

## Abstract

Vaccine hesitancy has become a pivotal consideration in assessing society’s readiness to accept recommended vaccination programs. The role of vaccination as a preventive measure during great epidemic challenges cannot be overestimated. On the other hand, the overwhelming flow of misinformation and attitudes resulting from denialism may have a profoundly harmful effect on the acceptance of preventive interventions. The adult Vaccine Hesitancy Scale (aVHS) is a result of efforts to develop a tool that will be relevant to the views about vaccination in the general adult population. It was derived from the Vaccine Hesitancy Scale (VHS), initially developed by researchers attempting to assess the opinions and attitudes of parents. This study’s main aim was to determine the reliability and validity of the Polish version of aVHS (PL-aVHS). We have also analyzed whether the scale can feasibly predict the COVID-19 vaccination status of respondents. The analysis was performed on data originating from a computer-based web-interviewing (CAWI) survey of 2008 adult Internet users. It included the analysis of internal consistency, test-retest reliability, and hypotheses testing. Exploratory (EFA) and confirmatory factor analyses (CFA) were performed on the subsets generated by randomly splitting the initial survey data. We have found that the scale has excellent internal consistency (Cronbach α = 0.935), acceptable levels of inter-item bivariate correlations, and good test–retest reliability (interclass correlation coefficient, ICC = 0.843). The EFA revealed that the tool has a two-factor latent structure; however, similar loadings of item 10 to both factors spoke for its exclusion from the model. Two extracted factors were responsible for 68.90% of the variance after rotation based on the maximum likelihood method. The CFA showed that the best fit of the model to measurement data was obtained for the two-factor model after excluding item 10. All seven fit indexes calculated in the analysis suggested at least an acceptable fit. In conclusion, the assessment of the PL-aVHS revealed good reliability and validity of the instrument. Furthermore, we have obtained similar EFA results as reported for the English version of the tool. Finally, to our knowledge, it is one of a few tools available in Polish for the measurement of vaccine-related attitudes.

## 1. Introduction

The SAGE Working Group on Vaccine Hesitancy defined vaccine hesitancy (VH) as “the delay in acceptance or refusal of vaccination despite the availability of vaccination services” [1]. Experts agree that VH is a complex and context-specific concept with many determinants. Probably the most popular model of the determinants of VH is the “3 Cs” model. It distinguishes three categories of determinants: complacency, convenience, and confidence [1]. Complacency means that the perceived risk of disease which can be prevented with a given vaccine is low, and vaccination is not treated as an unavoidable preventive measure. Convenience is a determinant associated with availability, affordability, willingness to pay, accessibility in terms of geographical circumstances, and finally, the ability to understand information about the vaccination. Confidence is based on the conviction that vaccines are effective and safe, as well as trust in the health care system and the policymakers making decisions about needed vaccines. In 2016, an extended 3C model of VH was proposed consisting of four categories [2]. ‘Calculation’ was the fourth new category in this 4C model. Thomson et al., based on the review of forty-three studies, developed a taxonomy of determinants of vaccine uptake consisting of 5 categories (“5 As”), including ‘access’, ‘affordability’, ‘awareness’, ‘acceptance’, and ‘activation’ [3]. Finally, recently Geiger et al. redefined and extended the components of vaccination readiness to seven items (7Cs), including ‘confidence’, ‘complacency’, ‘constraints’, ‘calculation’, ‘collective responsibility’, ‘compliance’, and ‘conspiracy’ [4]. Based on the 7Cs, they also developed the scale for assessing vaccine readiness, available in 7- and 21-item versions [4].

So far, several tools have been developed to assess VH or other constructs reflecting respondents’ attitudes towards vaccination, such as vaccine confidence or acceptance [2,5,6,7,8]. They consist of 4 [2] to 20 items [7]. Only the Global Vaccine Confidence Index (GVCI), used in the global study encompassing 67 countries, has a unidimensional structure [2]. Other instruments have two [9] to 5 subscales [7]. Furthermore, in 2018, Betsch et al. proposed a questionnaire assessing five psychological antecedents of vaccination [10].

Based on an extensive literature review, Larson et al. designed a survey tool encompassing a 10-item scale designed to assess parents’ vaccine hesitancy (VHS) [11]. This scale was later validated by Shapiro et al. among a large sample of Canadian parents [9]. They reported that the two-factor structure best explained the data from the survey. The factors identified with exploratory factor analysis were ‘lack of confidence’ and ‘risks.’ The score calculated based on the scale significantly correlated with respondents’ vaccine attitudes. VHS subscales were associated with vaccination refusals [9].

The generalized version of the VHS was developed and validated in the UK population by Luyten et al. [12]. These authors identified two subscales within the scale: lack of confidence in the need for vaccines and aversion to the risk of side effects. Exploratory factor analysis revealed that one of the items (item 10, asking about opinion of vaccines for diseases that are no longer common) loaded evenly to both factors and was excluded from the scale. Furthermore, they also reported that hesitancy measured with a generalized version of the scale was significantly associated with respondents’ characteristics, but these associations were rather low [12].

A modified version of the VHS for the assessment of adults’ attitudes towards vaccination was also proposed by Akel et al. [13]. They performed a series of three surveys (two in the USA and one in China), demonstrating the good internal consistency of their tool. Although this team maintained a 10-item scale, they also reported that the exclusion of item 10 resulted in higher internal consistency of the scale. An aVHS score ≥25 was taken to indicate that the given respondent was vaccine-hesitant. The aVHS scores were significantly associated with the prevalence of COVID-19 and flu vaccine acceptance. Also, other researchers developed modified versions of the VHS targeted either to use in the general population [14] or the assessment of hesitancy related to the use of specific types of vaccines, e.g., human papillomavirus vaccine (HPV) [15]. Furthermore, it also seems that cultural adaptations to French and Arabic are planned [16].

Our study’s main aim was to assess the reliability and validity of the aVHS adapted to Polish. Furthermore, the relationships between the aVHS score, health literacy, and attitudes towards vaccination against COVID-19 were assessed. According to the review of Sallam et al., COVID-19 vaccine acceptance based on the survey performed in January-February 2021, was only 50.8%. In other countries of Central and Eastern Europe, this rate ranged from 37.8% in Serbia to 73.3% in the Czech Republic [17]. Relatively low acceptance of COVID-19 vaccination justifies the need for a reliable instrument to measure the level of vaccine hesitancy in the Polish population.

The English version of the aVHS developed by Akel et al. was used as a source for the Polish translation [13]. An EFA was used to check the Polish version’s latent structure and a CFA was used to assess the fit of relevant models to data originating from a computer-based web-interviewing (CAWI) survey on a representative sample of adult Internet users.

## 2. Materials and Methods

### 2.1. Survey

The CAWI survey was performed on a representative sample of 2189 adult Internet users in late November and early December 2021. The survey was conducted by Ogólnopolski Panel Badawczy, a company specializing in public opinion and marketing research, and selected as a result of the obligatory tender bidding procedure for public organizations such as universities in Poland [18]. The company maintains an Internet Panel of 150,000 active registered respondents [19].

It is estimated that, in 2021, 83.6% of adults in Poland used the Internet at least once a week [20]. This percentage corresponds to about 24,000,000 Internet users aged 18–74. Assuming a fraction of 0.5 and a confidence level of 0.95, the sampling error was 2.1% at this population size. The stratified proportional sampling method was applied to obtain a sample corresponding to the structure of the population of Internet users in Poland concerning age, gender, level of education, place of residence, and Nomenclature of Territorial Units for Statistics (NUTS) 1 region.

The study received consent from the Bioethical Committee of the Jagiellonian University (Decision No 1072.6120.99.2020 from 23 April 2020, with amendments). Respondents were invited through an Internet panel to join the survey, received information about the study’s objectives, and were asked to provide their agreement before accessing the online questionnaire.

### 2.2. Questionnaire

The survey was based on a questionnaire consisting of 94 individual items. It included several validated tools: the 6-item European Health Literacy Survey Questionnaire (HLS-EU-Q6 [21], the 15-item General Conspiracy Belief Scale (GCBS), the 10-item e-Health Literacy Scale (eHEALS) [22], the 7-item General Anxiety Depression scale, the 5-item Future Anxiety Scale, items asking about adherence to preventive measures related to the COVID-19 pandemic and about attitudes toward vaccination against COVID-19, about opinions associated with the individual and the country-level situations during the pandemic, three items exploring beliefs in conspiracy theories related to the pandemic, a set of items asking about health behaviors, and nine socio-demographic questions. Finally, the Polish version of the 10-item adult Vaccine Hesitancy Scale (aVHS) and the 7-item Vaccine Conspiracy Belief Scale were also included. As our study is focused on validating the Polish version of the aVHS, only this scale and the HLS-EU-Q6 and items used for testing hypotheses are presented in detail.

The HLS-EU-Q6 is a short version of the 47 item tool developed by the team of the European Health Literacy Survey Project [23]. Due to the size of the questionnaire and the range of covered themes, we decided to use the short version of the questionnaire. The responses to the six items can be selected from a scale of very difficult to very easy. To the response options, values from 1 to 4 are assigned. If the respondents are not able to provide a response according to this scale or believe that the topic covered in the individual items is not relevant to their situation, they can select the option ‘I don’t know/not applicable.’ Such a response is treated as a missing value. The total HLS-EU-Q6 score is calculated as a mean of six individual scores only if there is not more than one missing value. Based on the score, the respondents are divided into three categories based on the level of HL: inadequate (score from 1 to 2), problematic (score from >2 to 3), and sufficient (score >3 to 4).

The aVHS is a modified version of the VHS scales developed by Larson et al. [11] and validated several years later by Shapiro et al. [9]. At least two teams developed modified English versions of the VHS scale for use in the general adult population or in relation to vaccines recommended for adults [12,13]. In our study, the version prepared by Akel et al. was applied [13]. This version of the aVHS consists of ten items. The responses to the items may be provided according to the 5-point Likert scale, from strongly disagree to strongly agree. Individual scores assigned to these responses span from 1 to 5. The answers to three items (item 5, 9, 10) are scored in reverse order. The total score may range from 10 to 50, with greater values indicating a higher level of vaccine hesitancy.

The survey questionnaire also included an item asking about respondents’ vaccination status against COVID-19. The respondent could select the following response options: ‘already vaccinated or in the course of vaccination’, ‘intent to vaccinate’, ‘not decided about vaccination’, and ‘not vaccinated and no intent to vaccinate.’

### 2.3. Cultural Adaptation of aVHS (PL-aVHS)

There are at least two English versions of the VHS tool adapted for use in the general adult population [12,13]. The authors obtained consent to adapt the aVHS, developed and validated by Akel et al., to Polish (personal communication from Abram L. Wagner from the Department of Epidemiology, University of Michigan, Ann Arbor, MI, USA, serving as a contact person for the team of authors).

We followed the WHO guidelines for transcultural adaptation of research instruments [24]. Two native-Polish-speaking persons with medical or public health professional backgrounds developed forward translations of the aVHS. The translation process was guided by striving to generate a conceptual equivalent instead of a word-for-word translation. Translators also observed the recommendation of using expressions relevant to Polish cultural contexts. Professional language or jargon was avoided.

The expert panel consisted of six persons, including two medical doctors with a specialty in pediatrics, one medical doctor specialized in internal medicine and public health, and four others with nursing, public health and nutrition, sociology, and linguistic backgrounds, respectively. The panel expressed their opinions about both translations, and the final version of the Polish version of the tool was established by consensus. The differences between the version of the scale targeted at the general adult population and the adapted earlier version for parents (an ongoing survey on parents of children with chronic diseases) based on the VHS validated by Shapiro et al. [9] were analyzed. It was agreed that item 4 in the English version developed by Akel et al. [13], requiring the respondent’s opinion about vaccines recommended by the CDC, would equate to physicians’ recommendations in the Polish version. The phrasing of this item proposed by Luyten et al. [12] was also considered. However, it was obvious that the equivalent to the recommendation originating from ‘governmental’ sources or the CDC does not function in public opinion in Poland, and physicians, mainly general practitioners, are the main source of such recommendations, especially in relation to the adult population.

Two independent translators, with English as their mother language, prepared the backward translation of the agreed scale version. They did not know the original questionnaire and were not professionally involved in medicine or public health. The original and back-translated versions of the scale were compared with an emphasis on terms critical for the topic of the instrument or parts that could be particularly sensitive to distortion during the translation process.

The scale was piloted in a group of 21 respondents representing diversified gender, age, and education level. In the pilot group, 47.6% (*n* = 10) were women. 80.1% (*n* = 17) lived in large cities, 9.5% (*n* = 2) lived in smaller cities and 9.5% (*n* = 2) lived in rural areas. 57.1% (*n* = 12) had university Bachelors or Masters degrees, 38.1% (*n* = 8) had completed secondary education, and 4.8% (*n* = 1) had completed post-secondary non-university education. 47.6% (*n* = 10) were single, and 42.9% (*n* = 9) were married. As for vocational status, 41.7% (*n* = 10) were employees, 12.5% (*n* = 3) were self-employed, and 12.5% (*n* = 3) were retired. They received paper forms with additional fields to provide feedback on aspects relevant to cognitive interviewing, including their thoughts when selecting the response options to the scale’s items, their motivations for selecting a given response, and terms or expressions that were not fully understandable. The expert panel members involved in the piloting phase further discussed the initial cognitive interviewing feedback. How the items were formulated did not raise reservations among participants in piloting. Only one participant in the pretesting phase declared that the expression “…diseases that are no longer common” is not fully understandable. However, the expert panel decided that one isolated voice did not justify changing the relevant item, especially since the expression had been repeated in popular media reporting the arguments of antivaccination movements. The original English and culturally adapted Polish versions of the aVHS are included in the Supplementary Material File: Table S1.

### 2.4. Statistical Analysis

IBM SPSS v.28 and IBM SPSS Amo 28 (IBM Corp. Armonk, NY, USA) software was used for the statistical analysis. Descriptive statistics were calculated for variables included in the analysis: absolute and relative frequencies for categorical variables and means, and standard deviations (SD) for continuous numerical variables. The comparison of the subgroups’ scores based on the aVHS was carried out with the Kruskal–Wallis test.

The Cronbach α coefficient was used to assess the internal consistency of the PL-aVHS. Coefficient values between 0.7 and 0.9 were taken to indicate good, and ≥0.9 as showing excellent internal consistency. A Guttman split-half coefficient was also calculated. A value of 0.8 was needed to determine sufficient internal consistency. The percentage of respondents who obtained a score of 10 was used to assess the floor effect, and a score of 50 points was used for the ceiling effect.

The test-retest reliability indicates the temporal stability of the tool. It was assessed based on the results of a scale filled in twice in the interval of 1–2 weeks by the same respondents. In this study, the aVHS was repeated after two weeks by 50 respondents. The mean and single item intraclass correlation coefficients (ICC) were calculated based on a two-way mixed model. Mean ICC reflects stability averaged across all raters, and single case ICC corresponds with the stability of an idealized single rater [25]. Usually, the mean ICC is higher than the single case ICC. According to the guidelines for the interpretation of mean ICCs, values <0.40 show poor, 0.40–0.59–fair, 0.60–0.74 good, and 0.75–1.00 excellent.

The Kaiser–Meyer–Olkin test was applied to check the adequacy of the sample size concerning the number of items included in the instrument. Following Hutcheson and Sofroniou, values >0.7 were expected to confirm adequacy [26]. In turn, Barlett’s test of sphericity was used to analyze the factorability of the data. Finally, the correlation matrix was explored to assess multicollinearity. It was assumed that a pair of items would not correlate much higher than 0.8 [27].

The construct validity of the scale was assessed based on hypotheses testing. The scores generated based on the PL-aVHS were evaluated among the subgroups showing various attitudes and practices toward vaccination against COVID-19. Furthermore, the correlation between the PL-AVHS score and the health literacy score was analyzed.

The EFA factoring was performed to analyze the latent variables responsible for the variance of the measure. The extraction of latent factors was conducted with the maximum likelihood method. The first dataset was obtained after initial survey data randomly splitting was used for the EFA. The communalities values were assessed before developing the EFA. It was assumed that the communality score would not be less than 0.2 [28]. The extraction of factors was guided by the Kaiser criterion requiring that the extracted factor’s eigenvalue equal at least 1.0. A scree plot was also generated to check how many factors should be retained. Varimax orthogonal rotation was used for the extraction of the principal factors. Factor loading <0.3 was assumed to be a suppressing and >0.4 a stable value [27,29]. It was also assumed that cross-loading of items should not be significant; the expected ratio of loadings was assumed as <75%. It was also expected that the extracted factors should have at least three items fulfilling relevant criteria. Finally, it was also expected that the retained factor would be responsible for at least 50% of the total variance [30].

The second dataset generated after the random splitting of the initial survey data was used to perform confirmatory factor analysis (CFA). The CFA’s main aim was to verify the factor structure of the PL-aVHS resulting from the EFA. The fit of the proposed factorial model and the estimation of the construct’s effects on the measured variables was assessed. The CFA was based on the estimation method of maximum likelihood. The CFA was conducted for the two-factor model resulting from the EFA for the Polish sample after inclusion and removal of item 10, as well as for the one-factor model reported by some researchers analyzing the latent structure of the tool developed originally by the WHO SAGE Working Group on Vaccine Hesitancy for parents [31]. The goodness-of-fit of the model was assessed based on several fit coefficients: the chi2-to-degrees-of-freedom ration (CDFR), the root-means-square error of approximation (RMSEA), the normed fit index (NFI), the comparative fit index (CFI), the goodness-of-fit index (GFI), the adjusted GFI according to degrees of freedom (AGFI), and the Tucker and Lewis Index (TLI). Based on the available literature, the expected values of fit indexes were as follows: for CDFR–<2.0, for NFI ≥0.90, for CFI >0.95, for GFI ≥0.85, for AGFI ≥0.80, for TLI ≥0.90 and for RMSEA <0.04 for good and 0.05–0.08 for acceptable feet [32,33,34]. It was decided that to confirm the goodness-of-fit of the data to the factor structure, at least four adequacy coefficients should follow reference levels.

## 3. Results

### 3.1. Characteristics of the Study Sample

The characteristics of the study samples and two subsets generated through the random splitting of the initial survey data are presented in Table 1. The mean age (standard deviation, SD) of respondents was as follows: in the whole sample, 44.10 (SD = 15.25), in subset 1, 44.26 (SD = 15.32), and subset 2, 43.95 (SD = 16.19).

### 3.2. Internal Consistency

The analysis has not revealed a significant floor (2.5%) or ceiling (1.1%) effect. The Cronbach α coefficient of 0.935 and the Guttman split-half coefficient of 0.950 indicated excellent internal consistency of the instrument. Bivariate correlations ranged between 0.401 and 0.810. Two of the bivariate correlations surpassed, to some degree, the expected level of 0.8; for items 1 and 3 (0.810) and item1 and 7 (0.806) (Table 2). We retained the relevant items as these correlations were not considerably above the suggested level. The correlation of individual items to the total score was between 0.6 (for item 9) and 0.848 (for item 7) (Table 3).

The values of Cronbach’s alpha after excluding individual items were lower for all items apart from items 9 and 10, for which coefficients were slightly higher (0.937 and 0.936, respectively) (Table 3). The two-week mean ICCs of the aVHS was 0.843 (95%CI: 0.724–0.911, *p* < 0.001), indicating excellent stability. The single item ICC was 0.728 (95%CI: 0.567–0.836, *p* < 0.001).

### 3.3. Exploratory Factor Analysis

The value of the Kaiser–Meyer–Olkin test was 0.947, so the sample size was adequate to conduct the EFA. Also, Barlett’s sphericity test confirmed the correlation matrix’s factorability (chi^2^ = 8642.08, *p* < 0.001). The communality scores were between 0.310 and 0.776.

**Figure 1:**
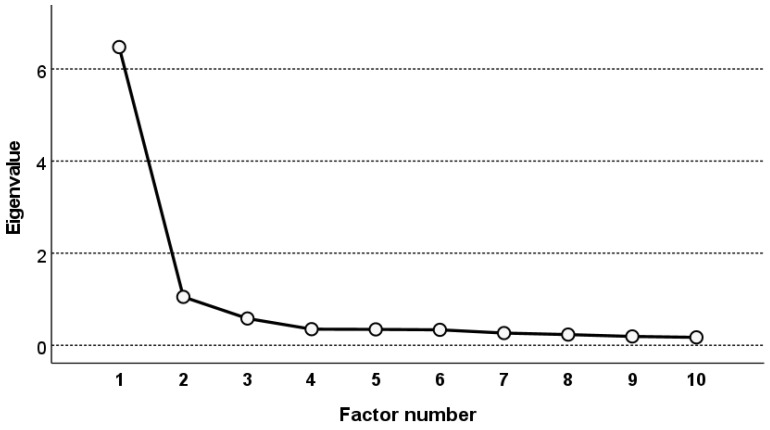
Scree plot.

The EFA indicated that the model valid for the sample consists of two factors (Table 4, Figure 1). The two factors explained 75.26% of the total variance. Their initial eigenvalues for the two factors were 6.48 and 1.05; after rotation, they were 4.68 and 2.21, respectively. The two factors extracted after rotation explained 68.90% of the variance.

Minimum factor loading after rotation was 0.503 for item 10 in relation to factor 2 (Table 5). The cross-loading for factor 10 reached the level of 80%. It was 5% above the expected level of 75% for the maximum ratio of loadings between factors; however, item 10 was one of three factors loading to factor 2. Therefore, it was retained in the two-factor model of the scale. The two factors resulting from the EFA were identified as ‘Confidence’ and ‘Risks’ analogically to the factors’ names proposed by Shapiro et al. in relation to the assessment of the original Vaccine Hesitancy Scale for parents [9].

### 3.4. Hypotheses Testing

Hypotheses testing was used to test construct validity. It was hypothesized that the aVHS score would be higher for those who were not vaccinated or were doubtful about vaccination against COVID-19. Another hypothesis used in this part of the analysis stated that the aVHS score would be higher among respondents with lower rather than higher HL. The scores for the two subscales and the whole scales were calculated for the groups of respondents distinguished based on their attitude toward vaccination against COVID-19 (Table 6). Post-hoc analysis revealed that the aVHS scores and subscores were significantly lower among the subgroup of respondents who were already vaccinated compared to all three remaining subgroups. The scores were significantly higher among those who were not vaccinated and were not going to get vaccinated than among the respondents who expressed the intent to get vaccinated (Table 6).

As hypothesized, the respondents with a sufficient level of HL had significantly lower levels of VH than those with inadequate or problematic HL (Table 6). The total PL-aVHS score was also significantly lower among those with problematic rather than inadequate HL.

### 3.5. Confirmatory Factor Analysis

**Figure 2:**
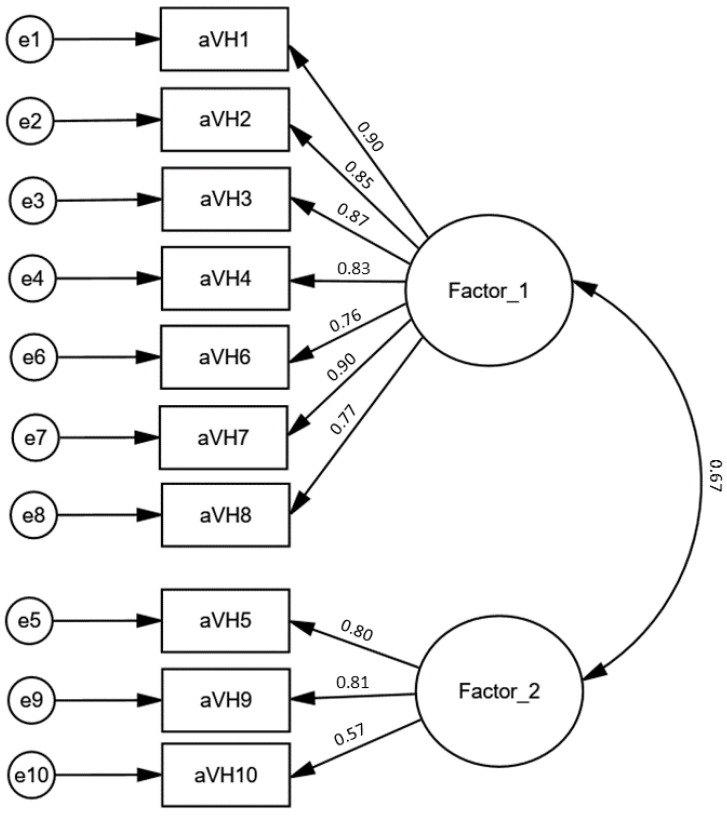
The CFA measurement model for the Polish version of the adult Vaccine Hesitancy Scale (PL-aVHS).

The CFA model developed for the 10-item PL-aVHS is presented in Figure 2. The results of fitting analysis of the two-factor models for the versions of the scale consisting of ten and nine items (after removing item 10) and for two unidimensional models of the versions of the scale with ten and nine items are shown in Table 7.

The CFA showed that two-dimensional models better fit the measurement data. Furthermore, after excluding item 10, the two-factor model was superior to the model including all ten items. The fit indexes for the two-factor model of the scale with nine items showed good or at least acceptable fit apart from the CDFR. NFI was 0.986, GFI 0.979, AGFI 0.964, CFI 0.989, TFI 0.985, and RMSEA (90%CI) 0.054 (0.043–0.064).

## 4. Discussion

This study reports the results of the assessment of the validation of the version of the VHS developed for the assessment of VH in the general adult population which has been translated to Polish (PL-aVHS). English versions of the VHS scale modified for use in general adult population were earlier prepared by two teams [12,13]. The Polish version of the scale results from the cultural adaptation of the aVHS proposed by Akel et al. [13]. The cultural adaptation followed the guidelines published earlier by the WHO [24]. EFA and CFA were performed on two datasets obtained through the random splitting of the data from the CAWI survey of 2186 adult Internet users. Our analysis showed that the Polish version of the aVHS has excellent internal consistency (Cronbach α = 0.935). The bivariate correlations also revealed adequate convergent and criterion validity (correlation 0.401–0.810). Two-week ICC showed, in turn, very good tool stability (0.843). An EFA showed that the scale has a two-factor latent structure; two factors accounted for 68.90% of variance after rotation. As item 10 loaded similarly to both factors, it was excluded from the final two-factor model. The two-factor structure was reported earlier by both Akel et al. and Luyten et al. [12,13]. The two factors established based on the results of EFA were, by analogy to factors distinguished by Akel et al. [13], identified as ‘Confidence’ (items 1–4, 6–8) and ‘Risks’ (item 5,9).

A CFA was performed on the second dataset resulting from the random splitting of the survey’s data. We have assessed the fit of four models of the aVHS: two one-factor models including 10 and 9 items (after exclusion of item 10) and two-factor models also including 10 and 9 items. The fit index of the two-factor models showed a decidedly better profile as 6 of the 7 indexes used in the analysis reached at least an acceptable level. The two-factor model with nine items was superior to the two-factor model with ten items. CDFR was the only index outside the recommended range for an acceptable model fit. It is usually emphasized that chi^2^ statistics and CDFR are affected by large samples [35,36]; in the case of our study, the CFA was carried out on a sample of 1107 respondents. Usually, it is expected that CDFR is lower than 2.0, but some authors also recommend that the acceptable level of the CDFR span from 2.0 to 5.0 [37]. In the case of the two-factor model of the PL-aVHS with nine items, CDFR was 4.187, so according to a more liberal approach, we reached an acceptable level. Similar CFA results were reported by Luyten et al. for their version of the scale, including nine items (with RMSEA of 0.075 and CFI of 0.90) [12]. They excluded item 10 (“I do not need vaccines for diseases that are not common anymore”) for the same reason as in our EFA; it loaded to a comparable extent to both latent factors. Although Akel et al. did not conduct CFA for their scale, they found that its internal consistency was improved after excluding item 10.

We have also conducted hypotheses testing, assuming that the aVHS score would be associated with the level of HL and respondents’ attitudes or status regarding vaccination against COVID-19. Indeed, both the total aVHS score and two subscores were significantly higher for those with inadequate and problematic HL than for those with sufficient HL. Furthermore, the respondents vaccinated against COVID-19 or intending to undergo vaccination had higher total aVHS scores in both subscores. During the COVID-19 pandemic, many studies were also performed in Poland to assess attitudes and practices related to COVID-19 vaccination. Most of these studies evaluated the hesitancy towards or the acceptance of COVID-19 vaccination based on singular items. Vaccinations against COVID-19 began in Poland in December 2020. In March 2021, Sowa et al. assessed the acceptance of COVID-19 vaccination among Polish citizens based on responses to one question asking about readiness to undergo vaccination if the vaccine was available right away [38]. The multivariable analysis of survey data revealed that apart from socio-demographic variables, the readiness to be vaccinated depended on the conspiracy index, attitudes to the threat of the coronavirus, compliance with other preventive measures, the fitness index, the social distance index, and concerns about the side effects of the COVID-19 vaccine [38]. In another study, Dziedzic et al. analyzed vaccine hesitancy to receive the booster dose of the COVID-19 vaccine in convenience samples of health professionals and students from two academic centers [39]. Their study assessed the willingness to receive vaccine booster doses based on one item on a 5-point Likert scale. Interestingly, in their sample, as many as 17.6% of respondents rejected the possibility of receiving the booster dose, and 7.9% were hesitant. Kuciel et al. assessed the acceptance of the COVID-19 vaccine among pregnant and lactating women and mothers of young children in Poland [40]. They also based their analysis on responses to a single question about willingness to be vaccinated. The Authors reported a strong association of confidence in receiving the COVID-19 vaccine with trust in the government, health professionals, and scientific authorities. Finally, Jastrzębska et al. assessed the relationship between knowledge about the pandemic and willingness to get vaccinated with the COVID-19 vaccine among medical students in Poland [41]. Their study was performed in Poland’s first months of COVID-19 vaccination. Interestingly, knowledge about the transmission routes of the new coronavirus was not statistically associated with being vaccinated in this group. Additionally, in this study, the research team specifically developed a questionnaire asking about the respondents’ various opinions on vaccination. Attitudes toward the COVID-19 vaccination were extensively researched in different samples of respondents in Poland. However, various research teams usually used tools that had not been validated or even single items to ask about acceptance or willingness to get vaccinated. Such a situation supports our efforts to deliver a validated and reliable tool, enabling the assessment of vaccine hesitancy in the Polish adult population.

### Limitations

One of the important limitations of online surveys is the exclusion of the part of the population not using the Internet and other electronic sources of information. In the case assessing VH in the adult population, those remaining on other side of the digital divide are likely to present with a specific profile of opinions about vaccination. This group would likely require special attention. The validity of surveys based on the CAWI technique is growing every year, even in countries like Poland, where the number of regular Internet users has been rather low for many years. However, according to recent statistics, the rate of adults who use the Internet at least once per week reached 83% in 2021 [20]. So, the number of potential respondents excluded from online surveys decreases yearly. Still, there is a risk that the elderly or those with low incomes are underrepresented.

With CAWI surveys, it is always a risk that respondents do not pay sufficient attention to the tasks of filling in the questionnaire. In this study, we used the professional service of a company experienced in opinion and research studies, and appropriate measures to assure the quality of the questionnaire were taken, e.g., those passing through the questionnaire too quickly were excluded from the study.

The validation of the PL-aVHS was conducted during a time of great challenges associated with the novel coronavirus. When assessing the results of measuring VH in the exposed population, one can expect that public awareness about vaccinations would be improved. On the other hand, it is obvious that the COVID-19 pandemic was accompanied by the enormous growth of radical opinions stemming from denialism and propelled by a flood of misinformation. As a result, for a significant part of society, the preventive measures against COVID-19 became an issue of political or emotional motivations. It is not fully clear how the radicalization of opinions in this area could influence the responses to items included in the aVHS.

To our knowledge, the aVHS is the first tool assessing VH adapted to Polish. On one hand, this is an added value of the efforts reported in this paper; on the other hand, we could not base hypotheses testing on the correlation with scores obtained from different instruments designed to measure hesitancy, confidence, or acceptance of vaccines.

## 5. Conclusions

This study showed that the Polish version of the aVHS is a reliable and valid instrument for measuring VH. Furthermore, its results are significantly related to the level of general health literacy. Finally, it may be used to predict the attitude toward vaccination with vaccines recommended for the adult population. In times of great epidemic challenges, when vaccination is one of the critical preventive measures, such an instrument is essential for anticipating the effectiveness of planned public health interventions. It should be underlined that test-retest reliability showed very good scale stability. A rapid review of the literature indicates that the assessment of the level of vaccine hesitancy has rarely been undertaken in Poland. The adaptation of the adult version of the VHS was not reported earlier for Polish adults. We believe that the availability of a reliable instrument to assess VH will improve the quality of studies conducted in Poland’s vaccinology field.

## Figures and Tables

**Table 1:** Characteristics of the study sample and subsets used for the exploratory and confirmatory factor analysis.

Variable	Variable Categories	All Respondents (*n* = 2189)		Subset 1 (*n* = 1082)		Subset 2 (*n* = 1107)	
		%	*n*	%	*n*	%	*n*
Gender	Female	51.2	1121	51.7	559	50.8	562
	Male	48.8	1068	48.3	523	49.2	545
Place of residence	Rural	37.87	829	36.9	399	38.8	430
	urban below 20,000 inhabitants	12.84	281	14.1	153	11.6	128
	urban 20,000–100,000 inhabitants	20.15	441	20.1	217	20.2	224
	urban 100,000–200,000 inhabitants	8.59	188	9.2	100	7.9	88
	urban 200,000–500,000 inhabitants	8.73	191	7.9	86	9.5	105
	urban above 500,000 inhabitants	11.83	259	11.7	127	11.9	132
Education	lower than secondary	13.8	301	13.2	143	14.3	158
	secondary vocational	24.4	535	23.5	254	25.4	281
	Secondary	35.7	781	38.5	417	32.9	364
	University	26.1	572	24.8	268	27.5	304
Net monthly household income	not more than 500 PLN	1.74	38	2.0	22	1.4	16
	501–1000 PLN	5.25	115	5.3	57	5.2	58
	1001–1500 PLN	8.77	192	9.2	100	8.3	92
	1501–2000 PLN	14.80	324	15.5	168	14.1	156
	2001–3000 PLN	21.52	471	21.3	231	21.7	240
	3001–4000 PLN	12.97	284	13.4	145	12.6	139
	more than 4000 PLN	13.57	297	12.9	140	14.2	157
	not revealed	21.38	468	20.2	219	22.5	249
Vocational status	employee	50.43	1104	50.8	550	50.0	554
	self-employed or farmer	7.26	159	6.2	67	8.3	92
	retired or on a disability pension	21.06	461	20.8	225	21.3	236
	high school or university student	4.80	105	5.4	58	4.2	47
	vocationally passive incl. unemployed	9.82	215	10.0	108	9.7	107
	a part-time job or other	6.62	145	6.8	74	6.4	71
Marital status	single	22.70	497	23.1	250	22.3	247
	married	53.86	1179	52.9	572	54.8	607
	in partnership	1.23	27	13.0	141	12.7	141
	widowed	3.97	87	3.6	39	4.3	48
	divorced or separated	6.58	144	13.80	80	14.30	64

Abbreviations: PLN–Polish zloty.

**Table 2:** Bivariate correlations of PL-aVHS items.

Item	Item 1	Item 2	Item 3	Item 4	Item 5	Item 6	Item 7	Item 8	Item 9
item 2	0.791								
item 3	0.810	0.774							
item 4	0.769	0.737	0.749						
item 5	0.479	0.512	0.479	0.451					
item 6	0.688	0.687	0.685	0.653	0.486				
item 7	0.806	0.799	0.772	0.783	0.525	0.681			
item 8	0.695	0.690	0.715	0.699	0.428	0.652	0.712		
item 9	0.459	0.487	0.478	0.447	0.661	0.475	0.483	0.401	
item 10	0.544	0.500	0.498	0.490	0.512	0.391	0.512	0.420	0.496

**Table 3:** Means, item-to-total correlations, and Cronbach’s alphas after removing specific items from the Polish version.

Item	Mean after Removing an Item	The Variance of the Scale after Removing an Item	Item-Factor Correlation	Squared Item-Factor Correlation	Cronbach α after Removing an Item	Initial Communalities
item 1	22.57	56.080	0.843	0.776	0.924	0.776
item 2	22.48	56.132	0.834	0.737	0.924	0.737
item 3	22.57	55.870	0.830	0.744	0.924	0.744
item 4	22.52	56.870	0.802	0.702	0.926	0.702
item 5	21.91	58.342	0.625	0.520	0.935	0.520
item 6	22.21	57.423	0.745	0.593	0.929	0.593
item 7	22.55	55.654	0.848	0.768	0.923	0.768
item 8	22.55	57.911	0.746	0.616	0.929	0.616
item 9	21.76	57.791	0.600	0.497	0.937	0.497
item 10	22.30	58.822	0.596	0.410	0.936	0.410

**Table 4:** Total variance explained by the two-factor latent structure of the scale.

Factor	Initial Eigenvalues			Sum of Squared Loading after Extraction			Sums of Squared Loadings after Rotation		
	Total	% of Variance	Cumulated % of Variance	Total	% of Variance	Cumulated % of Variance	Total	% of Variance	Cumulated % of Variance
1	6.48	64.75	64.75	6.17	61.74	61.74	4.68	46.83	46.83
2	1.05	10.51	75.26	0.72	7.17	68.90	2.21	22.08	68.90
3	0.58	5.82	81.07						
4	0.35	3.50	84.57						
5	0.35	3.45	88.02						
6	0.34	3.36	91.38						
7	0.27	2.65	94.04						
8	0.23	2.32	96.36						
9	0.19	1.92	98.28						
10	0.17	1.72	100.00						

**Table 5:** Factor loadings extracted with the maximum likelihood method and rotated with the varimax method.

Item	Factor 1	Factor 2
item 1	0.844	0.318
item 2	0.798	0.366
item 3	0.820	0.327
item 4	0.804	0.299
item 5	0.277	0.790
item 6	0.684	0.366
item 7	0.818	0.365
item 8	0.750	0.272
item 9	0.271	0.746
item 10	0.407	0.503

**Table 6:** The differences in the subscores and total PL-aVHS score between subgroups distinguished according to COVID-19 vaccination status and the level of HL.

Grouping Variable	Number of Respondents	‘Confidence’ Subscore Mean (SD)	K-W Test H Statistic (*p*-Value)	‘Risks’ Subscore Mean (SD)	K-W Test H Statistic (*p*-Value)	Total PL-aVHS Score Mean (SD)	K-W Test H Statistic (*p*-Value)
Status of vaccination against COVID-19							
subgroup 1	724	13.75 (4.78) ^2,3,4^	397.21 (<0.001)	7.46 (2.53) ^2,3,4^	316.34 (<0.001)	21.21 (6.47) ^2,3,4^	443.63 (<0.001)
subgroup 2	66	17.68 (4.00) ^1,3,4^		9.61 (1.76) ^1,4^		27.29 (4.80) ^1,4^	
subgroup 3	73	20.32 (3.61) ^1,2^		10.29 (1.55) ^1^		30.60 (4.02) ^1^	
subgroup 4	208	23.18 (5.89) ^1,2^		11.07 (2.30) ^1,2^		34.25 (7.15) ^1,2^	
Category of HL							
subgroup 1	49	18.98 (7.29) ^3^	18.31 (<0.001)	9.53 (2.87) ^3^	12.37 (0.002)	28.51 (9.46) ^2,3^	18.73 (<0.001)
subgroup 2	450	16.36 (5.88) ^3^		8.66 (2.59) ^3^		25.02 (7.79) ^1,3^	
subgroup 3	402	15.33 (6.29) ^1,2^		8.18 (3.05) ^1,2^		23.51 (8.68) ^1,2^	

^1,2,3,4^–numbers showing the subgroups with significant differences of the total score or subscore (within the column) assessed by post-hoc test with Bonferroni correction for multiple comparisons; HL–health literacy. Subgroups distinguished according to the status of COVID-19 vaccination: subgroup 1: already vaccinated or in the course of vaccination, subgroup 2: intention to vaccinate, subgroup 3: not decided about vaccination, subgroup 4: not vaccinated and no intent to vaccinate. Subgroups according to the level of HL: subgroup 1–inadequate, subgroup 2–problematic, subgroup 3–sufficient.

**Table 7:** The fitting results of three models of the PL-aVHS.

Indexes	Threshold Index Levels	One Factor Model (10 Items)	One Factor Model (9 Items)	Two-Factor Model (10 Items)	Two-Factor Model (9 Items without Item 10)
CDFR	<2.0 (*p* > 0.05)	17.016 (<0.001)	18.077 (<0.001)	5.47 (<0.001)	4.187 (<0.001)
NFI	Acceptable: ≥0.90 to <0.95, good: ≥0.95	0.927	0.937	0.977	0.986
GFI	Acceptable: ≥0.90 to <0.95, good: ≥0.95	0.904	0.917	0.966	0.979
AGFI	Acceptable: ≥0.90 to <0.95, good: ≥0.95	0.849	0.861	0.946	0.964
CFI	Acceptable: 0.90–0.95, good: >0.95	0.931	0.940	0.981	0.989
TLI	Acceptable: 0.90–0.95, good: >0.95	0.911	0.920	0.975	0.985
RMSEA (90%CI)	Acceptable: <0.08 to 0.05, good: <0.05	0.120 (0.112–129)	0.124 (0.115–0.134)	0.064 (0.055–0.073)	0.054 (0.043–0.064)

Abbreviations: CDFR–chi^2^ to degrees of freedom ratio (*p*-value), NFI–Bentler–Bonett normed fit index, GFI–the goodness of fit index, AGFI–adjusted GFI CFI–Bentler comparative fit index, TLI–Tucker and Lewis Index, RMSEA (90%CI)–root mean square error approximation (90% confidence limit).

## Data Availability

The data are not publicly available due to privacy and ethical restrictions. The authors did not include in the information about the study provided to the participants that public access to the data obtained during the survey may be considered. Access to the data will be granted on a case-by-case basis for a justified request after receiving consent from the Bioethical Committee at Jagiellonian University.

## Supplementary Materials

The following supporting information can be downloaded at: https://www.mdpi.com/article/10.3390/vaccines10101666/s1, Table S1: English and Polish versions of the aVHS.
