# Supplementary material for: The Assessment of the Reliability and Validity of the Polish Version of the Adult Vaccine Hesitancy Scale (PL-aVHS) in the Context of Attitudes toward COVID-19 Vaccination
Source: Vaccines (Basel). 2022 Oct 6;10(10):1666. doi: 10.3390/vaccines10101666 (PMC9609930; doi:10.3390/vaccines10101666)
Supplement: Supplementary file 1 [file vaccines-10-01666-s001.zip › vaccines-1922491-supplementary.pdf]

Table S1. Items of the questionnaire.

Item	English version	Polish version
item 1	Vaccines are important for my health.	Szczepienia są ważne dla mojego zdrowia.
item 2	Vaccines are effective.	Szczepienia są skuteczne.
item 3	Being vaccinated is important for the health of others in my community.	Zaszczepienie się jest ważne dla zdrowia innych osób w moim otoczeniu.
item 4	All routine vaccinations recommended by the CDC are beneficial.	Wszystkie rutynowe szczepienia zalecane przez lekarzy są korzystne.
item 5	New vaccines carry more risks than older vaccines.	Nowe szczepionki niosą ze sobą więcej zagrożeń niż starsze.
item 6	The information I receive about vaccines from the CDC is reliable and trustworthy.	Informacje o szczepionkach, które otrzymuję od pracowników ochrony zdrowia są rzetelne i wiarygodne.
item 7	Getting vaccines is a good way to protect me from disease.	Szczepienia to dla mnie dobry sposób zabezpieczenia się przed chorobami.
item 8	Generally, I do what my doctor or healthcare provider recommends about vaccines for me.	Jeśli chodzi o szczepienia, to zazwyczaj kieruję się zaleceniami lekarza lub innego pracownika ochrony zdrowia.
item 9	I am concerned about serious adverse effects of vaccines.	Obawiam się poważnych skutków ubocznych szczepionek.
item 10	I do not need vaccines for diseases that are not common anymore.	Nie potrzebuję szczepień przeciwko chorobom, które nie są już częste.
